# CRISPR-based diagnostics for infectious diseases: mechanisms, advancements and clinical transformation prospects

**DOI:** 10.3389/fcimb.2026.1769226

**Published:** 2026-02-24

**Authors:** Zhenzhen Pan, Ling Xu, Zihao Fan, Yaling Cao, Feng Ren

**Affiliations:** Beijing Institute of Hepatology, Beijing Youan Hospital, Capital Medical University, Beijing, China

**Keywords:** Cas proteins, CRISPR, infectious diseases, molecular diagnostics, point-of-care

## Abstract

Infectious diseases continue to pose significant global public health challenges, necessitating the development of rapid, sensitive, specific, and field-deployable diagnostic platforms. The discovery of Clustered Regularly Interspaced Short Palindromic Repeats (CRISPR) and CRISPR-associated proteins (Cas) has revolutionized genome editing and concurrently enabled a new generation of molecular diagnostic tools. Leveraging the inherent trans-cleavage activities of Cas enzymes, platforms such as SHERLOCK (Specific High-sensitivity Enzymatic Reporter unLOCKing) and DETECTR (DNA Endonuclease-Targeted CRISPR Trans Reporter) have emerged, combining target recognition precision with reporter systems to achieve ultra-sensitive detection of pathogen-specific nucleic acids. This review systematically examines the mechanistic foundations of CRISPR diagnostics, synthesizes recent advancements in infectious disease applications, evaluates their advantages in sensitivity, specificity, operational simplicity, and multiplexing capacity, and critically analyzes current implementation barriers and future translational pathways.

## Graphical Abstract

**Figure:**
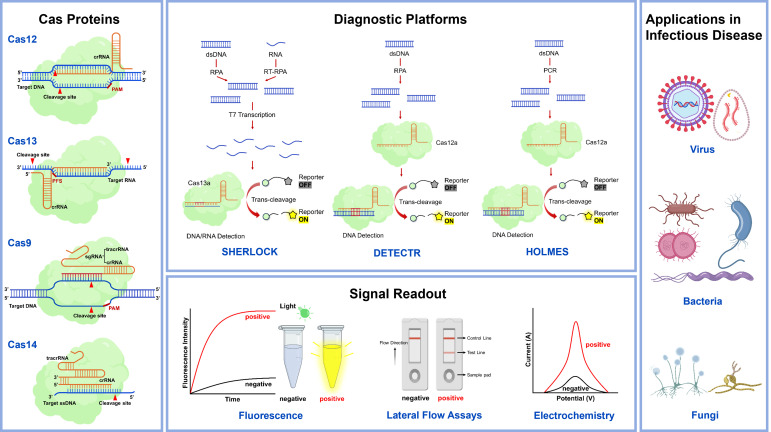

## 1 Introduction

### 1.1 Current status and challenges in infectious disease diagnostics

Accurate and rapid identification of infectious pathogens forms the cornerstone for effective infection control, precision treatment protocols and outbreak prevention (Standing up to infectious disease, 2019). Clinical diagnostic practices currently rely on three primary methodologies: pathogen isolation and cultivation, polymerase chain reaction (PCR)-based molecular platforms, and immunological assays. While these approaches constitute the modern diagnostic framework for infectious diseases and perform adequately in routine settings, their limitations become starkly apparent in managing emerging/re-emerging pathogens and point-of-care testing (POCT) scenarios.

Specifically, the “gold standard” pathogen cultivation method, though capable of providing antibiotic susceptibility profiles, suffers from prolonged turnaround times (hours to weeks), limited sensitivity, and biosafety hazards (Cao et al., 2024). PCR-based molecular diagnostics have significantly improved speed and sensitivity but remain reliant on sophisticated laboratory infrastructure, lack discrimination between viable and non-viable pathogens, and face constraints in multiplexing capacity and cost efficiency (Shin et al., 2024). Immunological methods (antigen/antibody detection) offer operational simplicity but are compromised by the “window period” phenomenon, host immune status variations, and inherent sensitivity/specificity limitations, often yielding only qualitative results (Liu et al., 2025). These limitations collectively highlight the urgent clinical need for new diagnostic technologies, particularly those enabling rapid, field-deployable, highly sensitive detection without requiring complex equipment.

In summary, despite the value of traditional methods, their shortcomings in speed, sensitivity, operational convenience, equipment dependency, and field deployment capabilities drive global research efforts toward next-generation diagnostic technologies. CRISPR-Cas-based molecular diagnostics have emerged in this context, aiming to fill these technological gaps.

### 1.2 Discovery of CRISPR-Cas systems

The development of CRISPR-Cas systems represents a monumental breakthrough in modern biology. First observed in 1987 by Japanese researcher, the system was initially identified through its distinctive short palindromic repeat sequences (Ishino et al., 1987). In the early 2000s, Spanish researcher Mojica discovered homology between these sequences and bacteriophage genes, proposing CRISPR might function as a prokaryotic adaptive immune system (Mojica et al., 2005). This hypothesis was experimentally validated in 2007 by Barrangou’s team, who demonstrated bacterial acquisition of specific phage immunity through CRISPR mechanisms (Barrangou et al., 2007).

CRISPR-Cas functionality proceeds through three phases: adaptation (spacer acquisition), expression (crRNA biogenesis), and interference (target interference) (van Beljouw et al., 2023). A transformative breakthrough occurred in 2012 when Charpentier and Doudna’s groups engineered a chimeric single-guide RNA (sgRNA) by fusing tracrRNA and crRNA, demonstrating programmable DNA cleavage by the Cas9 nuclease *in vitro*, this is a seminal finding that catalyzed the CRISPR genome editing revolution (Jinek et al., 2012). In 2013, the research group led by Feng Zhang first demonstrated the utility of CRISPR-Cas9 for precise gene editing in eukaryotic cells (Cong et al., 2013). This was followed in 2015 by the discovery of the Cas12a system, which broadened the range of targetable sequences (Zetsche et al., 2015). The subsequent introduction of the RNA-targeting Cas13 system in 2016 further diversified the targeting possibilities (East-Seletsky et al., 2016). Then, in 2017, the development of base editing technology enabled precise nucleotide alterations without requiring double-strand DNA breaks (Gaudelli et al., 2017). Together, these breakthroughs significantly enriched the versatility and precision of genomic engineering tools.

### 1.3 Expanding CRISPR-Cas into diagnostic applications

Since the initial adaptation of the CRISPR-Cas system for nucleic acid detection in 2016 (Pardee et al., 2016), numerous CRISPR-based diagnostic platforms have been developed for the detection and diagnosis of both infectious and non−infectious diseases (Kaminski et al., 2021). The revolutionary potential of CRISPR-Cas, first demonstrated in gene editing, is now extending vigorously into the field of molecular diagnostics, positioning it as a promising alternative to PCR in many applications (Mohammadhassan et al., 2022).

The transformation of CRISPR-Cas systems from genome editing tools to diagnostic platforms primarily relies on the trans-cleavage activity of Class 2 effector proteins (e.g., Cas12, Cas13). This property enables CRISPR systems to function as ultra-sensitive molecular detection tools by cleaving reporter molecules non-specifically upon target recognition.

CRISPR diagnostics operate through crRNA-guided target recognition coupled with trans-cleavage activity. When target nucleic acids are present in a sample, the Cas protein-crRNA complex binds to the sequence, activating trans-cleavage function that cleaves fluorescently or biotin-labeled reporter molecules, generating detectable signals (Li et al., 2018). Compared to conventional PCR and ELISA, CRISPR-based diagnostics offer advantages in operational simplicity, superior specificity, and attomolar (aM) sensitivity, making them particularly suitable for point-of-care testing (POCT).

### 1.4 Objectives

This review aims to systematically summarize the latest research progress in applying CRISPR technology to infectious disease detection, with in-depth analysis of its technical principles, methodological innovations, and clinical application value. The article will first elucidate the core mechanisms of CRISPR systems as molecular diagnostic platforms. Subsequently, it comprehensively reviews specific applications of this technology in detecting various pathogens, including viruses, bacteria, and fungi. Finally, the review will objectively discuss current technical challenges and outline future development directions. By systematically organizing the current status and trends in this field, this review provides a comprehensive technical reference for researchers and promotes the clinical translation and application of CRISPR-based detection technologies.

## 2 Mechanisms and platforms of CRISPR-based diagnostics

### 2.1 Common Cas proteins used for diagnosis

**Figure 1:**
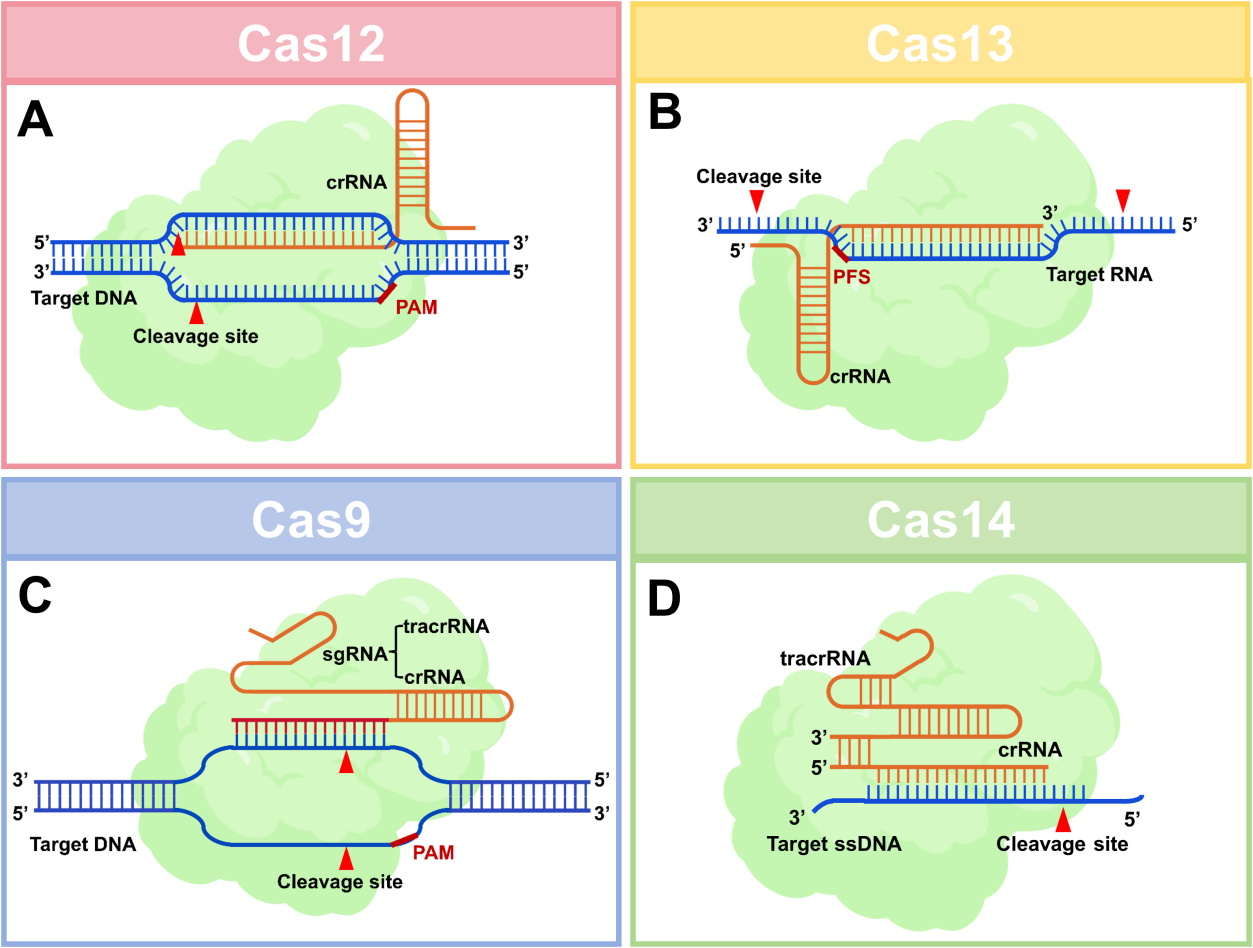
The cleavage mechanisms of four Cas proteins used for diagnosis. **(A)** Cas12, upon recognizing and cleaving its target DNA, is activated and subsequently performs indiscriminate cleavage of any surrounding single-stranded DNA molecules, enabling cascading signal amplification. **(B)** Cas13, following target RNA recognition and cleavage, becomes activated and conducts indiscriminate cleavage of any surrounding single-stranded RNA molecules, facilitating highly sensitive RNA detection. **(C)** Cas9 functions as a programmable double-stranded DNA endonuclease whose cleavage activity is strictly confined to the target sequence itself, lacking any signal amplification capability. **(D)** Cas14 directly recognizes and cleaves single-stranded DNA (ssDNA), subsequently activating indiscriminate ssDNA cleavage activity, making it particularly suitable for the direct detection of ssDNA targets. Schematics employ a simplified representation for comparative clarity, reflecting functional domains rather than precise tertiary structures. Created with BioGDP.com (Jiang et al., 2025).

CRISPR-Cas systems are categorized into two classes based on effector complex composition: Class 1 systems (Types I, III, IV) utilize multi-subunit effector complexes, while Class 2 systems (Types II, V, VI) employ single-protein effectors. Class 2 effectors demonstrate distinct advantages in molecular diagnostics due to their structural simplicity and enhanced engineering potential (Koonin and Makarova, 2019). Therefore, only Class2 effectors are introduced here. The cleavage mechanisms of the four Cas proteins are shown in **Figure 1**. And the distinct mechanisms, amplification dependencies, and POC suitability of Cas9, Cas12, Cas13, and Cas14 are concisely summarized in **Table 1**.

**Table 1:** Comparative analysis of major CRISPR-cas effector proteins in molecular diagnostics.

Cas proteins	Primary nucleic acid target	Collateral (trans-cleavage) activity	Signal amplification	Pre-amplification requirement	Suitability for portable (POC) detection	Primary diagnostic utility
Cas9	Double-stranded DNA (dsDNA)	Absent.	No intrinsic amplification. Relies on coupled reporter systems.	Mandatory. Requires highly sensitive pre-amplification (e.g., PCR) to generate detectable signal.	Low. Highly dependent on thermocycling and sophisticated instrumentation; not suitable for rapid field deployment.	Target enrichment and complex assay architectures (e.g., using dCas9 for capture). Not a core platform for rapid, amplification-coupled diagnostics.
Cas12	Double-stranded DNA (dsDNA)	Present.	Yes.	Mandatory for clinical sensitivity. Requires isothermal pre-amplification (e.g., RPA, LAMP).	High. Operates at moderate temperatures (≈37 °C); amenable to “one-pot” assay formats; mature platform with extensive validation (e.g., DETECTR).	Mainstream DNA detection (DNA viruses, bacterial genomics, human genetic variants).
Cas13	Single-stranded RNA (ssRNA)	Present.	Yes.	Mandatory for clinical sensitivity. Requires reverse transcription coupled with isothermal amplification (e.g., RT-RPA, RT-LAMP).	High. Operates at moderate temperatures; ideal for direct RNA virus detection (e.g., SHERLOCK).	Mainstream RNA detection (RNA viruses, gene expression markers).
Cas14	Single-stranded DNA (ssDNA)	Present.	Yes.	Typically required. May be circumvented for very high-copy-number ssDNA targets under optimized conditions.	Moderate with High Potential. Ultra-compact size favors reagent miniaturization, lyophilization, and multiplexing.	Emerging specialist for direct ssDNA virus detection and high-fidelity SNP genotyping without a denaturation step.

#### 2.1.1 Cas12a (Cpf1)

Representing Type V systems, Cas12a exhibits unique post-cleavage trans-cleavage activity, non-specifically cleaving single-stranded DNA (ssDNA) reporters upon target engagement. This property enables ultra-sensitive DNA detection (e.g., DNA viruses, SNP genotyping). Cas12a recognizes T-rich PAM sequences (5’-TTTN-3’), expanding targeting scope, and generates sticky ends post-cleavage, facilitating downstream cloning applications (Nouri et al., 2021).

#### 2.1.2 Cas13a (C2c2)

As a Type VI effector, Cas13a uniquely targets RNA rather than DNA. Similar to Cas12a, it activates collateral RNA cleavage upon target binding, making it ideal for RNA virus detection (e.g., SARS-CoV-2, dengue, Zika). Cas13a requires a protospacer flanking site (PFS) and its trans-cleavage activity underpins high-sensitivity RNA detection platforms (Gootenberg et al., 2017).

#### 2.1.3 Cas9

While Cas9 revolutionized genome editing, its lack of trans-cleavage activity limits direct diagnostic utility. Researchers circumvent this by coupling catalytically inactive dCas9 with signal output modules (e.g., fluorescent reporters) or using it for target enrichment, thereby enhancing detection sensitivity (Xin et al., 2022).

#### 2.1.4 Cas14 (Cas12f)

Cas14 represents a class of exceptionally compact RNA-guided nucleases (400–700 amino acids), with most Cas14 proteins being nearly half the size of the smallest Cas9 or Cas12 nucleases. Despite their small size, Cas14 proteins are capable of targeting and cleaving single-stranded DNA (ssDNA) without stringent sequence constraints. Furthermore, target recognition by Cas14 triggers collateral cleavage of ssDNA molecules, an activity that enables applications such as high-precision SNP genotyping (Cas14-DETECTR) (Nguyen et al., 2022).

#### 2.1.5 CRISPR-Cas variants

To overcome limitations of native systems—such as narrow target range, stringent assay conditions, and insufficient sensitivity or specificity—protein engineering and directed evolution have yielded CRISPR-Cas variants that advance diagnostic performance.

AapCas12b is a thermostable variant derived from Alicyclobacillus acidiphilus that maintains high activity at 60–65 °C. Elevated-temperature reactions not only accelerate the assay (to within 5–20 minutes) but also reduce nonspecific amplification (e.g., primer-dimer formation), thereby enhancing the signal-to-noise ratio and sensitivity. Platforms based on AapCas12b have been successfully deployed for rapid screening of pathogens like SARS-CoV-2 (Ali et al., 2022).

By leveraging a catalytically dead Cas9 (dCas9) mutant with retained DNA-binding capability, methods such as Bio-SCAN have been developed. This approach enables precise detection of the SARS-CoV-2 genome without requiring additional reporters, probes, enhancers, or complex readout devices. Incorporating variant-specific sgRNAs further allows efficient discrimination between SARS-CoV-2 variants (e.g., Alpha, Beta, and Delta) (Zhang et al., 2021).

These engineered variants are driving diagnostics toward higher sensitivity, specificity, ease of use, and broader application scenarios.

### 2.2 Major diagnostic systems

**Figure 2:**
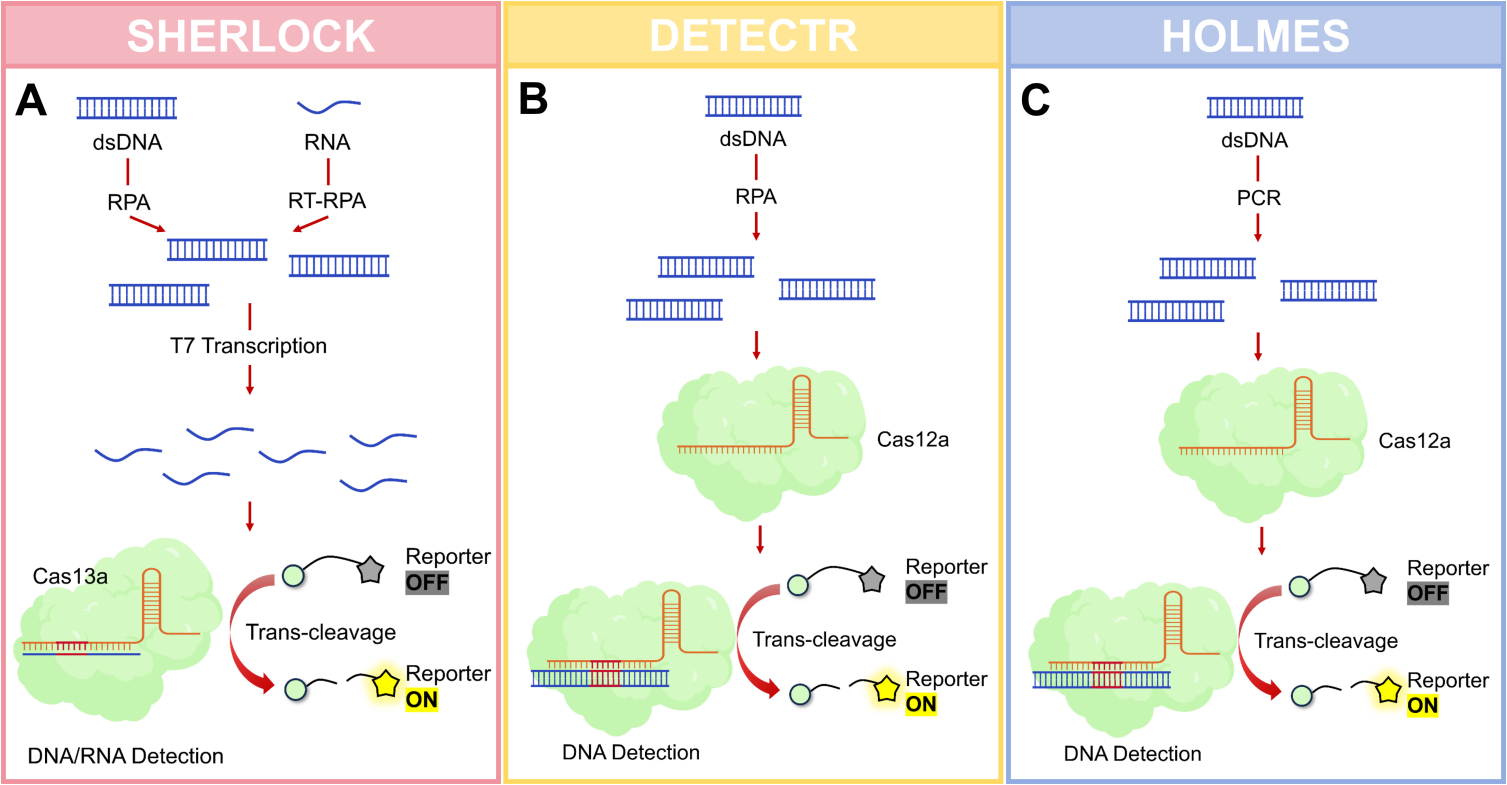
Major diagnostic systems. **(A)** Schematic of SHERLOCK: This platform utilizes Cas13 for the detection of RNA or DNA targets. The process typically involves an isothermal pre-amplification step (e.g., RPA or RT-RPA), followed by Cas13-mediated target recognition. Upon binding to the target RNA, activated Cas13 exhibits collateral cleavage of an added ssRNA reporter molecule, generating a fluorescent or colorimetric signal detectable via a fluorometer or lateral flow strip. **(B)** Schematic of DETECTR: This system employs Cas12a for the detection of DNA targets. Following isothermal amplification (e.g., RPA or LAMP), the Cas12a-gRNA complex binds to the target double-stranded DNA. This binding activates Cas12a’s nonspecific trans-cleavage activity, which degrades an ssDNA reporter to produce a measurable signal, commonly read out by fluorescence or lateral flow assay. **(C)** Schematic of HOLMES: HOLMES is designed for DNA detection but utilizes the thermostable Cas12 nuclease. Its key feature is operation at an elevated temperature (~60-65 °C), which accelerates the reaction, enhances specificity by reducing non-specific amplification, and often couples with LAMP for pre-amplification. Signal generation occurs via Cas12b’s target-activated collateral cleavage of an ssDNA reporter. Created with BioGDP.com (Jiang et al., 2025).

CRISPR-Cas-based molecular diagnostic platforms have rapidly evolved due to their exceptional sensitivity, specificity, and operational simplicity. SHERLOCK and DETECTR represent the two most prominent systems, leveraging the trans-cleavage activities of Cas13 and Cas12a respectively to establish new paradigms in nucleic acid detection. **Figure 2** shows three common diagnostic systems.

#### 2.2.1 SHERLOCK

Developed by Zhang’s group in 2017, this platform utilizes Cas13a/crRNA complexes to recognize specific RNA targets, activating collateral RNA cleavage of fluorescently labeled reporters. The workflow integrates reverse transcription-recombinase polymerase amplification (RT-RPA) or loop-mediated isothermal amplification (LAMP) for target pre-amplification, achieving aM sensitivity (Gootenberg et al., 2017). SHERLOCKv2 (2018) introduced multiplexing capabilities through orthogonal Cas13 variants (Cas13a/b/c) and Csm6 nucleases, coupled with lateral flow assay readout, significantly enhancing field applicability (Gootenberg et al., 2018).

#### 2.2.2 DETECTR

Pioneered by Doudna’s team in 2018, this platform employs Cas12a’s trans-cleavage activity on single-stranded DNA (ssDNA) reporters following target dsDNA recognition (Chen et al., 2018). Combined with RPA isothermal amplification, DETECTR completes detection within 30–60 minutes at aM sensitivity. Clinical validation demonstrated 100% concordance with PCR for HPV16/18 genotyping, establishing its diagnostic accuracy (Zhao et al., 2023).

#### 2.2.3 Other diagnostic platforms

HOLMES (HOur Low-cost Multipurpose Highly Efficient System) was developed by the Shanghai Institute of Life Sciences, the first-generation HOLMES utilized Cas12a for viral DNA detection and genotyping. HOLMESv2 introduced Cas12b, maintaining activity across 37-60 °C and improving thermal stability. This PCR-CRISPR hybrid system achieves 10 aM sensitivity through synergistic amplification and detection (Li et al., 2019). The operational characteristics, clinical validation status, and a critical appraisal of the strengths and limitations of SHERLOCK, DETECTR, and HOLMES are systematically consolidated in **Table 2**. This synthesis provides a foundational framework for evaluating their suitability in various diagnostic scenarios discussed in the following sections.

**Table 2:** Comparative performance and critical appraisal of major CRISPR-based diagnostic platforms.

Diagnostic platforms	Core assay characteristics				Critical performance appraisal					Positioning relative to PCR	
	Time-to-result	Readout method	Analytical sensitivity	Clinical validation status	Performance with clinical samples	Tolerance to inhibitors in crude samples	False-positive rate & specificity	False-negative rate & causes	Key practical limitations	Scenarios of superiority to PCR	Scenarios as a complementary tool to PCR
SHERLOCK	30–60 minutes	Fluorometer/Lateral Flow Assay	aM level, single-copy	Extensive.	Excellent for purified RNA.	Poor. RPA is highly susceptible to common clinical inhibitors, leading to false negatives.	Primary risk stems from nonspecific amplification by RPA.	High. 1) Incomplete removal of inhibitors; 2) Degradation of labile RNA targets during processing.	1) RNA lability imposes stringent sample handling; 2) Two-step format increases contamination risk; 3) Limited multiplexing capability.	Superior in speed and cost-effectiveness for rapid, qualitative screening of RNA targets in resource-limited settings.	1) Rapid Triage: High-throughput screening with PCR confirmation/quantification of positives; 2) Alternative Pathway: Useful for samples containing potent PCR inhibitors.
DETECTR	30–45 minutes	Fluorometer/Lateral Flow Assay	aM level	Extensive.	Robust for DNA targets.	Moderate. LAMP is more tolerant than RPA but remains vulnerable to high inhibitor concentrations.	Risk mainly from LAMP nonspecificity.	Moderate.1) Target concentration below detection limit; 2) Suboptimal reaction conditions.	1) PAM sequence requirement limits design flexibility; 2) Requires precise isothermal control; 3) Poor quantitative capability.	Superior in operational simplicity and turnaround time for field-deployable DNA screening.	1) Decentralized Testing: Enables rapid testing at primary care levels for patient triage; 2) Outbreak Surveillance: Facilitates early pathogen identification at outbreak epicenters.
HOLMES	40–60 minutes	Fluorometer/Real-time fluorescence	aM level	Rapidly expanding.	High-temperature incubation reduces nonspecific binding, requires precise temperature control.	Moderate to High. Elevated temperature may mitigate some inhibitor effects, conferring potential robustness.	Theoretically lowest.	Moderate. 1) Inaccurate temperature control impairing Cas12b activity; 2) Suboptimal sample processing.	1) Requires stable high-temperature (~60 °C) instrumentation; 2) Newer platform with less accumulated validation data; 3) Challenges in lyophilized reagent formulation.	Superior for rapid DNA tests requiring high specificity, leveraging its temperature-mediated fidelity advantage.	High-Fidelity Rapid Testing: Offers a faster, cost-effective alternative to ddPCR for SNP genotyping or discrimination of homologous sequences.
Reference: qPCR	1.5–2 hours	Real-time fluorometer	Single-copy, aM-fM level	Gold standard.	Highly robust.	High. Can be effectively managed with additives, sample dilution, and internal controls.	Extremely Low.	Low. Effectively monitored via internal amplification controls.	1) High cost and dependence on specialized instruments/operators; 2) Long turnaround time, unsuitable for point-of-care; 3) Requires standard laboratory infrastructure.	Serves as the benchmark for comparison.	–

Furthermore, continuous innovation has led to the emergence of derived platforms such as the Cas14-based DETECTR-CAS14, designed specifically for detecting single-stranded DNA viruses and single-nucleotide polymorphisms (SNPs) (Harrington et al., 2018), and the CRISPR-Dx platform, capable of simultaneous detection of both DNA and RNA pathogens (Yoshimi et al., 2022). These ongoing developments enrich the CRISPR diagnostic toolkit, offering expanded options for pathogen detection, genotyping, and mutation analysis.

### 2.3 Signal readout modalities

**Figure 3:**
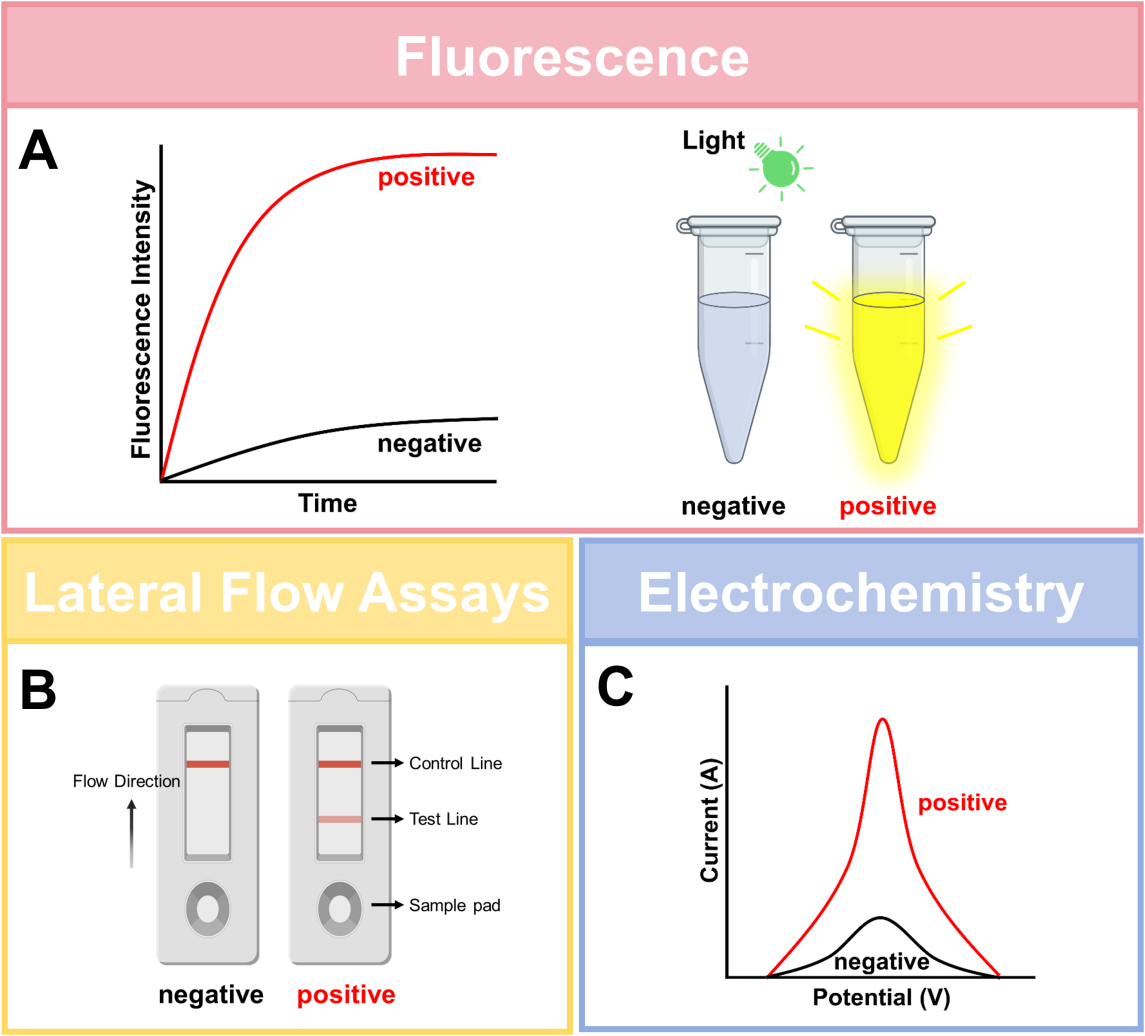
Three widely-used signal readout modalities. **(A)** Fluorescence-based detection: A quencher-fluorophore-labeled reporter oligonucleotide is cleaved by the activated Cas enzyme’s collateral activity (e.g., Cas12/13/14), resulting in fluorescence recovery. **(B)** Lateral flow assay: Cleaved or intact reporter molecules, often labeled with gold nanoparticles or colored latex beads, are captured at specific lines on a nitrocellulose strip. The appearance of both control **(C)** and test (T) lines indicates a valid assay and positive result, respectively. **(C)** Electrochemical detection: CRISPR-mediated recognition and cleavage events are transduced into measurable electrical changes (e.g., current, voltage, impedance) via functionalized electrodes or field-effect transistors. Created with BioGDP.com (Jiang et al., 2025).

Signal readout strategies represent a critical component of CRISPR-based diagnostics, enabling visualization and quantitative analysis of detection results. Multiple approaches have been developed to meet diverse application requirements. The three common signal readout modalities are shown in **Figure 3**.

#### 2.3.1 Fluorescence detection

The most widely used and sensitive method relies on fluorescently labeled reporter molecules. Upon target activation, Cas proteins (e.g., Cas12/13) cleave fluorescence-quenched (FQ) reporter probes, releasing fluorescent signals. These signals are quantifiable via real-time qPCR instruments, microplate readers, or portable fluorometers. Platform-specific examples include SHERLOCK’s RNA reporters (e.g., 6-FAM/UU/3IABkFQ) and DETECTR’s ssDNA reporters (e.g., 6-FAM-TTATT-3BHQ) (Gootenberg et al., 2017). While offering quantitative capabilities and high-throughput potential, this method requires sophisticated equipment, limiting point-of-care utility.

#### 2.3.2 Lateral flow assays

This approach advances field deployment by using biotin/FAM-labeled ssDNA reporters. Cas-mediated cleavage releases FAM-tagged fragments that bind anti-FAM antibodies on test lines (T-line), while biotinylated moieties interact with streptavidin on control lines (C-line). Integrated into SHERLOCKv2 and DETECTR platforms, this method enables visual interpretation within 30 minutes using paper-based strips, achieving fM-level sensitivity (Myhrvold et al., 2018). Though less sensitive than fluorescence methods, its portability and simplicity make it ideal for resource-limited settings.

#### 2.3.3 Other readout modalities

Other emerging readout modalities include electrochemical sensors and colorimetric assays. Electrochemical sensors integrate CRISPR reactions with electrode systems, detecting target nucleic acids by measuring changes in current, voltage, or impedance. For example, when an ssDNA reporter is immobilized on an electrode surface, activation of Cas12 cleaves the reporter, altering the electron transfer properties of the electrode and producing a measurable electrical signal. This method offers high sensitivity, ease of miniaturization, and system integrability (Dai et al., 2019). Colorimetric methods rely on visual color changes mediated by nanomaterials (e.g., gold nanoparticles) or enzymatic reactions (e.g., horseradish peroxidase, HRP). For instance, activated Cas13 can cleave an HRP-conjugated RNA reporter, modulating color intensity (Kellner et al., 2019). These emerging approaches are driving CRISPR diagnostics toward higher sensitivity, lower cost, and greater portability.

## 3 Applications in infectious disease detection

### 3.1 Viral infectious disease detection

#### 3.1.1 RNA viruses

Leveraging the Cas12a system, researchers have developed multiple innovative strategies for human immunodeficiency virus (HIV) detection. These include a CRISPR-Cas12a-PCHA method that activates palindromic-catalytic hairpin assembly for multiplex signal amplification, an amplification-free HIV DNA assay using gold nanoparticles (AuNPs) for dual signal enhancement, and a gel separation-based “one-pot” detection system (Zhao et al., 2022; Zhou et al., 2022; Uno et al., 2023). These approaches enable ultrasensitive detection down to the aM level and facilitate visual diagnosis within 30 minutes. Furthermore, an innovative strategy that converts CRISPR-mediated signals into glucometer readings shows promise for at-home point-of-care HIV testing (Li et al., 2023). Simultaneously, the Cas13a system enables RNA-targeted point-of-care screening via lateral flow strips, while an amplification-free digital CRISPR platform (STAMP-dCRISPR) provides precise viral load quantification comparable to RT-PCR, offering powerful tools for early HIV diagnosis and therapeutic monitoring (Nouri et al., 2023).

The integration of CRISPR-Cas12a/b systems with isothermal amplification has led to efficient detection platforms for hepatitis viruses that belong to RNA viruses. For hepatitis C virus (HCV), the engineered thermostable Cas12b system SPLENDID enables detection within 20 minutes at 67 °C (Nguyen et al., 2023). A single-tube visual platform reduces detection time to 15 minutes with 100 copies/μL sensitivity, while a lateral flow strip-based method achieves 10 copies/μL sensitivity in 30 minutes (Wang et al., 2022; Wei et al., 2024). For hepatitis D virus (HDV), the CRISPR-Cas13a system demonstrates superior performance with an 81.9% positive detection rate in clinical samples, significantly outperforming conventional molecular assays (Tian et al., 2023b). For hepatitis E virus (HEV), CRISPR-Cas13a-based methods achieve detection limits of 12.5 IU/mL and 200 IU/mL for fluorescence and lateral flow readouts, respectively, with a single-tube assay shortening detection time to 35 minutes while maintaining accuracy (Li et al., 2023).

Multiple CRISPR-based approaches have been developed for SARS-CoV-2 detection. Wei et al. employed entropy-driven amplification combined with CRISPR/Cas13a system, achieving 7.39 aM sensitivity via electrochemiluminescence signaling (Wei et al., 2023). Ma et al. developed a CRISPR/Cas12a-based assay integrated with surface-enhanced Raman spectroscopy (SERS), enabling amplification-free detection within 45 minutes with 200 copies/mL sensitivity in a single tube (Ma et al., 2023). Yang et al. integrated CRISPR/Cas13a with catalytic hairpin assembly, attaining 84 aM detection limit within 35 minutes while effectively discriminating SARS-CoV-2 from related viruses (Yang et al., 2023).

Furthermore, The Cas13a-based assay enables direct detection of Ebola virus RNA without amplification, achieving 175 copies/μL detection limit and completing workflow within 40 minutes at 37 °C without pre-amplification or centrifugation (Hang et al., 2022). Additionally, the Naval Medical University established a single-pot RT-RPA-CRISPR/Cas12a system targeting conserved regions across dengue virus serotypes, obtaining an impressive detection limit of 9.17 copies/μL (Zhang et al., 2025).

#### 3.1.2 DNA viruses

DNA virus detection primarily utilizes Cas12 proteins (e.g., Cas12a/b) through trans-cleavage of ssDNA reporters. For instance, a Cas12a/QDNB-based lateral flow assay (CQ-LFA) targeting the varicella-zoster virus ORF31 gene demonstrated a limit of detection (LOD) of 0.2 copies/μL, showing 100% concordance with qPCR results across 86 clinical vesicular samples without requiring nucleic acid extraction (Zhong et al., 2023). In CRISPR/Cas12a-based HPV-16 detection, a microfluidic fluorescence assay integrated with a transition-state molecular switch employs a dual screening mechanism to effectively suppress non-specific adsorption, achieving low-background and highly specific detection while distinguishing various HPV subtypes and base mismatches, with LODs of 7.64 pM (mean fluorescence intensity) and 9.91 fM (pixel count) (Sui et al., 2025).

The Cas13 protein can also be applied to DNA virus detection. One study reported a test strip method based on RAA-CRISPR/Cas13a for detecting HBV DNA in patients with low-level viremia, demonstrating a sensitivity of 10^1^ copies/μL and 100% specificity (Tian et al., 2023a). Zhang X et al. established a novel CRISPR-Cas13-based method for cccDNA detection by integrating sample pretreatment, amplification, and detection steps. Following rolling circle amplification (RCA) and nested PCR, the assay achieved detection of HBV cccDNA at a sensitivity as low as 1 copy/μL through fluorescence readout (Zhang et al., 2022).

### 3.2 Bacterial detection

#### 3.2.1 *Mycobacterium tuberculosis* and drug-resistant strains

Tuberculosis (TB) remains one of the deadliest infectious diseases globally, with multidrug-resistant TB (MDR-TB) posing substantial diagnostic challenges. Although the traditional phenotypic drug susceptibility testing (pDST) is the “gold standard”, it is time-consuming and delays treatment initiation. Researchers at Shenzhen Third People’s Hospital developed the CRISPR-Cas14a MTB RIF/INH platform for direct detection of rifampicin (RIF)- and isoniazid (INH)-resistant genetic mutations (e.g., rpoB, katG, and inhA genes) in clinical MTB isolates and sputum samples. This platform employs specific gRNAs to distinguish wild-type from mutant sites, integrating multiplex PCR amplification and CRISPR trans-cleavage reactions (Xiao et al., 2025). Beyond Cas14a, Cas12a and Cas13a systems have also been applied for MTB and drug-resistance gene detection, exhibiting high sensitivity and specificity (Ai et al., 2019; Sam et al., 2021).

#### 3.2.2 Methicillin-resistant *Staphylococcus aureus*

Methicillin-resistant Staphylococcus aureus (MRSA), a major pathogen in hospital- and community-acquired infections, derives resistance primarily from the mecA gene. Professor Wei Cheng’s team at the First Affiliated Hospital of Chongqing Medical University developed a CRISPR/Cas12a-based method combined with cross-priming amplification (CPA), achieving detection within 30 minutes with a LOD of 5 CFU/mL. Lateral flow strip visualization enables field deployment (Wu et al., 2023). Another study established an RPA-CRISPR Cas13a assay for rapid MRSA resistance gene detection (Gu et al., 2025).

#### 3.2.3 Salmonella

For Salmonella, a common foodborne pathogen, the EXPAR-CRISPR/Cas12a method targets the yfiR gene, achieving a 10 fM LOD on synthetic DNA and successfully identifying Salmonella-containing genomic DNA extracts (threshold: 1 pg/μL) within 1 hour. This approach offers speed, affordability, and high specificity (Lin et al., 2025). A centrifuge-driven microfluidic chip integrated with RPA-CRISPR enabled visual Salmonella detection with 1×10² CFU/mL sensitivity while preventing aerosol contamination (Chen et al., 2025).

#### 3.2.4 Neisseria gonorrhoeae

Neisseria gonorrhoeae antibiotic resistance is a growing concern, necessitating rapid diagnostics for sexually transmitted infection control. A team at the First Affiliated Hospital of Chongqing Medical University developed an RPA-CRISPR/Cas12a system for equipment-free *N. gonorrhoeae* detection within 1 hour, demonstrating 100% concordance with traditional culture methods (Tu et al., 2023).

#### 3.2.5 Other pathogen detection

In bacterial pathogen detection, an RAA-CRISPR/Cas13a assay targeting Brucella based on the BCSP31 gene achieved LOD of 100 copies/μL and 10¹ copies/μL for fluorescence and lateral flow readouts, respectively, both with 100% specificity (Liu et al., 2025). For Haemophilus influenzae (HI), the EFORCA platform integrated rapid lysis with RAA and CRISPR technologies, enabling extraction-free single-tube detection of bacterial suspensions at 50 CFU/mL within 30 minutes (Cao et al., 2025).

In antimicrobial resistance detection, Professor Feng Ren’s team established a CRISPR/Cas13a system for precise identification of carbapenem-resistant Klebsiella pneumoniae (CRKP), where the PCR-CRISPR method achieved an LOD of 1 copy/μL for KP, blaKPC, and blaNDM genes, while the RAA-CRISPR method reached 10¹ copies/μl (Feng et al., 2025).

For Mycoplasma pneumoniae, the novel TRACER technology enabled seamless integration of RPA amplification and CRISPR-Cas12b detection in a single tube through precise temperature control, achieving a detection sensitivity of 1 copy/μL (Zeng et al., 2025).

Furthermore, CRISPR has been extended to fungal detection. An ERA-CRISPR/Cas12a system targeting the ITS2 region of Candida albicans was optimized into a one-pot temperature-controlled assay, completing detection within 30 minutes with an LOD of 100 ag/µL. Its high specificity and closed-tube operation effectively prevent cross-reactivity and aerosol contamination (van Dongen et al., 2020).

These findings collectively demonstrate the broad clinical application prospects of CRISPR technology in pathogen diagnostics, particularly highlighting its unique advantages in enabling rapid, highly sensitive, and user-friendly point-of-care testing.

### 3.3 Multiplex detection

CRISPR systems enable multiplex detection—simultaneous identification of multiple pathogens or variants in a single reaction—which is critical for diagnosing coinfections, subtyping pathogens, and monitoring viral mutations. By combining distinct Cas proteins with corresponding crRNAs and reporters, parallel detection of DNA viruses, RNA viruses, and bacteria becomes feasible. Signal readout strategies for CRISPR-based multiplexing fall into three categories: (a) Multicolor Fluorescence Systems: Use reporters labeled with distinct fluorophores (e.g., FAM, HEX, Cy5), each paired with specific Cas-crRNA complexes. Multi-channel fluorescence detection enables differential analysis. (b) Lateral Flow Assays: Incorporate reporters tagged with FAM/biotin, binding to corresponding antibodies on test lines for visual multiplexing. (c) Electrochemical Sensors: Modify electrodes with target-specific molecules, differentiating analytes through electrical signal patterns (Yin et al., 2024).

The SEDphone platform (Hefei Institutes of Physical Science, CAS) integrates microfluidics with LAMP-CRISPR/Cas12a, achieving 45-minute simultaneous subtyping of H1N1, H3N2, H5N1, H7N9, and influenza B virus (IBV) with a 10 copies/μL LOD, readable via smartphone (Zhang et al., 2024). A portable centrifugal microfluidic system (POCMT) developed by Nanjing University combines nucleic acid extraction, RAA amplification, and CRISPR-Cas13a detection on a single chip, enabling 10-pathogen (e.g., Japanese encephalitis virus, Ebola, dengue, Zika) detection within 45 minutes with plasmid LODs of 1 copy/reaction (Yan et al., 2025).

In addition to enabling multiplex detection of diverse pathogens, CRISPR systems facilitate high-fidelity genotyping of single-nucleotide polymorphisms (SNPs). For instance, by precisely modulating crRNA concentrations (e.g., 10 nM crRNA1 and 3 nM crRNA2) to mitigate signal interference caused by trans-cleavage activity, researchers have successfully achieved triplex genotyping—discriminating wild-type, mutant, and heterozygous genotypes—of the rs4646536 SNP in the CYP27B1 gene, which plays a critical role in vitamin D metabolism (Cao et al., 2024).

## 4 Performance advantages and existing challenges

The application of CRISPR technology in infectious disease diagnostics demonstrates significant advantages over conventional methods. However, as an emerging technology, it still faces several critical challenges that must be addressed before achieving widespread clinical adoption.

### 4.1 Advantages

#### 4.1.1 High sensitivity

When coupled with isothermal amplification technologies like recombinase polymerase amplification (RPA) or loop-mediated isothermal amplification (LAMP), CRISPR assays achieve aM sensitivity, detecting even single-copy viral nucleic acids. For example, SHERLOCKv2 demonstrated a LOD of 1 copy/μL for Zika and dengue viruses, matching or surpassing traditional gold-standard methods like PCR (Gootenberg et al., 2017).

#### 4.1.2 High specificity

Guided by crRNA, CRISPR-Cas systems exhibit unparalleled specificity, enabling discrimination of single-nucleotide polymorphisms (SNPs). This facilitates precise differentiation of closely related pathogen species, subtypes, or drug-resistant mutations—such as distinguishing all 144 possible combinations of H1-H16 and N1-N9 influenza subtypes (Ackerman et al., 2020).

#### 4.1.3 Rapidity and simplicity

Traditional qPCR requires hours and specialized thermal cyclers. In contrast, CRISPR diagnostics integrated with isothermal amplification reduce total workflow time (from nucleic acid release to result interpretation) to 30–60 minutes. Simplified protocols minimize hands-on steps and reduce reliance on trained personnel.

#### 4.1.4 Point-of-care testing application

A hallmark advantage, CRISPR reactions operate at constant temperatures with results readable via portable fluorometers or naked-eye lateral flow dipsticks (LFD). This enables POCT in resource-limited settings like clinics, airports, and outbreak sites (Kellner et al., 2019). Recent platforms such as hippo-CORDS, PddCas, and CLIPON further advance viral POCT applications (Ackerman et al., 2020; Tang et al., 2022; Xue et al., 2023).

### 4.2 Challenges and limitations

Despite its promising advantages, the translation of CRISPR-based diagnostics into mature and reliable clinical products faces several critical challenges.

#### 4.2.1 Sample pretreatment

Efficient, rapid, and low-cost extraction of high-quality nucleic acids from complex clinical samples (e.g., sputum, blood, nasal swabs) remains a major bottleneck in achieving fully integrated “sample-to-result” systems. Inhibitors present in samples (such as hemoglobin and mucin) can significantly impair amplification efficiency and CRISPR reaction performance.

#### 4.2.2 Primer/crRNA design

Although CRISPR systems themselves are highly specific, the preceding isothermal amplification step requires carefully designed primers to avoid non-specific amplification. Similarly, crRNAs must be meticulously optimized to maximize on-target specificity and cleavage efficiency while minimizing off-target effects and non-specific signals. This imposes high demands on bioinformatics tools and design strategies (Chen et al., 2020).

#### 4.2.3 Reaction condition optimization

The activity of Cas enzymes is highly dependent on reaction conditions such as temperature, pH, and ion concentration (e.g., Mg²^+^). Developing Cas variants that maintain high activity and stability across a broad range of conditions, as well as producing lyophilized reagents suitable for storage and transport at ambient temperatures, is essential to ensure reliability and broad applicability (Teng et al., 2019).

#### 4.2.4 Cost and scalability

The current cost of recombinant Cas enzymes and synthetic crRNAs remains relatively high. Achieving large-scale production and application will require optimization of protein expression and purification processes, development of efficient lyophilization formulations, and miniaturization and cost reduction of detection devices.

#### 4.2.5 Standardization and quality control

Ensuring the reliability of CRISPR diagnostics necessitates building a comprehensive system for quality control, spanning from manufacturing to performance evaluation. On one hand, lot-to-lot consistency of the core Cas enzyme reagents is critical for maintaining stable assay performance. Manufacturing must implement standardized controls for each batch’s activity, purity, and contaminants to minimize variations in sensitivity or background signal caused by reagent variability, thereby ensuring long-term comparability of patient results. On the other hand, the field urgently requires standardized reference materials, such as accredited pseudoviral particles or synthetic biological samples with precisely defined concentrations. Such universal “yardsticks” enable objective comparison and independent verification of performance across different platforms, provide a basis for regulatory assessment and inter-laboratory result alignment, and ultimately empower clinicians to select and trust diagnostic products based on harmonized criteria.

#### 4.2.6 Regulatory approval and clinical validation

As *in vitro* diagnostic (IVD) devices, CRISPR-based tests must undergo stringent regulatory review (e.g., by the FDA, CE, or NMPA) (Mustafa and Makhawi, 2021). The clinical translation of CRISPR-based diagnostics follows distinct regulatory pathways, the choice of which determines a product’s intended use and evidentiary standard. Emergency Use Authorization (EUA) facilitates rapid deployment during public health emergencies, relying on relatively limited clinical data and intended for temporary screening purposes. In contrast, obtaining full *in vitro* diagnostic (IVD) approval requires evidence from large-scale prospective clinical studies, along with the establishment of a complete quality system and post-market surveillance. This pathway represents the ultimate goal for a product to be used as a routine diagnostic tool guiding long-term clinical decision-making. Clinicians must understand this hierarchical nature of regulatory approvals to critically evaluate the strength of evidence and the appropriate context for applying results from different products.

## 5 Future perspectives and conclusion

CRISPR technology, as a revolutionary molecular diagnostic tool, has demonstrated immense potential in infectious disease detection. Future development will focus on three directions: technological innovation, application expansion, and clinical translation.

### 5.1 Technological fusion and innovation

Future breakthroughs in this field are expected to arise from interdisciplinary integration. Primarily, the deep convergence with microfluidics technology is crucial. By integrating nucleic acid extraction, isothermal amplification, and CRISPR reactions onto a single chip, fully automated “sample-to-answer” diagnostic systems can be developed, which dramatically streamline operational procedures and minimize aerosol contamination (Xie et al., 2022). Secondly, paper-based sensors and wearable devices offer promising platforms for CRISPR diagnostics, potentially enabling low-cost, portable, and continuous health monitoring.

At the molecular level, the exploration and engineering of novel Cas proteins are central to expanding detection capabilities. For instance, compact effectors such as Cas14 and CasX—with their smaller molecular size, distinct PAM requirements, and efficient cleavage activity—provide new opportunities to develop more compact detection systems and target a broader range of genetic sequences (Harrington et al., 2018; Yang and Patel, 2019).

Furthermore, the incorporation of artificial intelligence (AI) is set to revolutionize experimental design. Machine learning algorithms can predict crRNA off-target effects, optimize guide RNA designs, and forecast reaction efficiency and specificity based on large experimental datasets, substantially shortening development timelines and enhancing detection reliability (Shokoohi et al., 2025).

### 5.2 Application expansion

The CRISPR diagnostic platform will continuously expand its boundaries. Spatially, its applications will extend from central laboratories to primary care facilities, home self-testing, outbreak sites, and resource-limited regions, becoming an affordable and accessible diagnostic tool to promote global health equity.

Furthermore, the scope of detection targets will extend far “beyond infectious diseases”. Leveraging its capacity to detect nucleic acid mutations and perform high-resolution genotyping, CRISPR technology is already being explored for multifaceted applications. These include not only cancer gene mutation profiling (e.g., in EGFR and KRAS) but also precise molecular diagnostics in malignancies where genetic and epigenetic characterization is critical for therapy, such as in acute myeloid leukemia (AML).This translational relevance is underscored by recent oncology reviews highlighting the importance of targeted and epigenetic analyses in AML treatment (SherkatGhanad et al., 2023). The platform’s utility further spans food safety monitoring (e.g., detection of foodborne pathogens and genetically modified ingredients) and environmental microbiology (Jolany Vangah et al., 2020). The versatility of the CRISPR platform thus positions it as a powerful and multifaceted tool for molecular detection across diverse fields.

### 5.3 Clinical transformation

The translation of advanced laboratory technologies into reliable commercial products is paramount for realizing their societal impact. This necessitates robust industry-university-research collaboration to advance key translational aspects, including large-scale production, lyophilization protocols, quality control, and cost management, thereby enabling stable and scalable manufacturing. However, this transition faces significant practical constraints. As outlined in **Table 3**, most CRISPR diagnostic technologies are currently transitioning from the laboratory validation to the translational development phase. This intermediate stage is characterized by persistent dependencies (e.g., on pre-amplification) and a lack of standardized validation, which directly explains the practical constraints hindering robust commercial manufacturing.

**Table 3:** CRISPR diagnostic platforms: current translational and regulatory status overview.

Stage / State	Key characteristics	Representative platforms/technologies	Key challenges and limitations
1. Basic Research & Technology Validation	1) Proof-of-concept and performance optimization in controlled laboratory settings. 2) Published in academic journals, demonstrating high analytical sensitivity and specificity.	1) Discovery and characterization of novel Cas variants (e.g., Cas14, CasΦ). 2) Proof-of-concept for novel detection mechanisms (e.g., dCas9-based assays).	1) Performance heavily reliant on idealized samples (purified nucleic acids). 2) Lack of standardized operating protocols and evaluation frameworks. 3) Not yet systematically validated on real-world clinical samples.
2. Translational Development & Preliminary Clinical Evaluation	1) Development of integrated, user-friendly workflows (e.g., one-pot assays, lateral flow strips). 2) Initiation of retrospective or small-scale prospective clinical studies.	1) Optimized platforms based on Cas12/13 (e.g., SHERLOCKv2, DETECTR). 2) Laboratory-developed tests (LDTs) for specific pathogens.	1) Persistent dependence on isothermal pre-amplification, introducing susceptibility to inhibitors and potential impacts on robustness. 2) Multi-center, large-scale clinical validation data remain limited. 3) Scalable manufacturing and batch-to-batch reagent consistency need to be established.
3. Regulatory Approval & Clinical Application	1) Authorization from regulatory bodies (e.g., FDA EUA, CE-IVD marking). 2) Deployment as IVD products in specific clinical scenarios.	1) FDA EUA-authorized CRISPR assays for SARS-CoV-2. 2) CE-marked HPV genotyping CRISPR tests.	1) Approvals are typically restricted to specific targets and sample types, limiting broader application. 2) Absence of internationally harmonized performance standards and reference materials, hindering result interoperability. 3) As novel methodologies, their cost-effectiveness and clinical utility require long-term real-world evidence.

Concurrently, securing regulatory approval (e.g., from the FDA, CE, or NMPA) and entering clinical markets makes the establishment of international consensus and standards an urgent priority. Efforts must focus on developing widely accepted SOPs, unified performance evaluation criteria (e.g., thresholds for sensitivity, specificity, and reproducibility), and shared reference materials for calibration and control. Such standardization is critical to overcome the current fragmented validation landscape and to provide a clear, efficient pathway for the evaluation and approval of diagnostic products, thereby accelerating the progress of regulatory science in this field (Mustafa and Makhawi, 2021).

### 5.4 Conclusion

In summary, CRISPR-based diagnostics represent a pivotal direction for the future of molecular testing. Their high sensitivity, specificity, speed, and portability hold paradigm-shifting potential for traditional infectious disease detection. Notwithstanding formidable challenges in sample preparation, reaction optimization, standardization, and regulatory approval, the considerable potential and continuous innovation of CRISPR technology foreshadow its expanding role in clinical practice and public health response. Through global collaboration across scientific and industrial communities, CRISPR is poised to provide core tools for building a more robust, adaptable, and equitable global health defense system.
